# Supplementary material for: Machine learning models based on remote and proximal sensing as potential methods for in-season biomass yields prediction in commercial sorghum fields
Source: PLoS One. 2021 Mar 25;16(3):e0249136. doi: 10.1371/journal.pone.0249136 (PMC7993797; doi:10.1371/journal.pone.0249136)
Supplement: Attachment — Submitted filename: Response to Reviewers.pdf [file pone.0249136.s003.pdf]

Response to reviewers

Dear Editor,

Thank you very much once again for considering my paper for publication in the journal PLOS ONE.

It's a pleasure for myself and on behalf of the co-author, to be able use the Editor and Reviewer constructive comments and suggestions to improve this manuscript.

I systematically went through the comments and suggestions, providing clarifications and better framing concepts that were not clearly detailed in the previous version of the manuscript. We thank Editor and Reviewers for the opportunity offered to better be useful to the scientific community.

Below, our answers are provided in italicized font preceded with the capital letter "R" followed by a column ":"

Cordially.

Bologna, March 10, 2021

Ephrem.

PONE-D-20-28051R1
Machine learning models based on remote and proximal sensing as potential methods for in-season biomass yields prediction in commercial sorghum fields
PLOS ONE

Dear Dr. Habyarimana,

Thank you for submitting your manuscript to PLOS ONE. After careful consideration, we feel that it has merit but does not fully meet PLOS ONE's publication criteria as it currently stands. Therefore, we invite you to submit a revised version of the manuscript that addresses the points raised during the review process.

Please address the over fitting issue raised by reviewer 1, in particular how the linear model parameters are obtained.

Please submit your revised manuscript by Apr 22 2021 11:59PM. If you will need more time than this to complete your revisions, please reply to this message or contact the journal office at plosone@plos.org. When you're ready to submit your revision, log on to https://www.editorialmanager.com/pone/ and select the 'Submissions Needing Revision' folder to locate your manuscript file.

Please include the following items when submitting your revised manuscript:

- A rebuttal letter that responds to each point raised by the academic editor and reviewer(s). You should upload this letter as a separate file labeled 'Response to Reviewers'.
- A marked-up copy of your manuscript that highlights changes made to the original version. You should upload this as a separate file labeled 'Revised Manuscript with Track Changes'.
- An unmarked version of your revised paper without tracked changes. You should upload this as a separate file labeled 'Manuscript'.

If you would like to make changes to your financial disclosure, please include your updated statement in your cover letter. Guidelines for resubmitting your figure files are available below the reviewer comments at the end of this letter.

If applicable, we recommend that you deposit your laboratory protocols in protocols.io to enhance the reproducibility of your results. Protocols.io assigns your protocol its own identifier (DOI) so that it can be cited independently in the future. For instructions see: http://journals.plos.org/plosone/s/submission-guidelines#loc-laboratory-protocols

We look forward to receiving your revised manuscript.

Kind regards,

Jie Zhang
Academic Editor
PLOS ONE

Journal Requirements:

Please review your reference list to ensure that it is complete and correct. If you have cited papers that have been retracted, please include the rationale for doing so in the manuscript text, or remove these references and replace them with relevant current references. Any changes to the reference list should be mentioned in the rebuttal letter that accompanies your revised manuscript. If you need to cite a retracted article, indicate the article's retracted status in the References list and also include a citation and full reference for the retraction notice.

Additional Editor Comments (if provided):

Reviewer 1 has a valid point on the fact that less number of samples the inputs can have a serious impact on the model generalisation, i.e. the over fitting problem. This is especially the case for linear model if the conventional MLR is used for finding model parameters. To cope this problem, principal component regression, or partial least square regression, or ridge regression should be used. The authors need to clarify how they obtained the linear model parameters.

R: We agree with the Editor on these issues of overfitting and multicollinearity, particularly in the linear model. Usually a few options are available in order to minimize the effect of multicollinearity and these can include: using models that are resilient to sizeable between-feature correlations, such as PLS, PCA, and other machine learning algorithms least squares. Alternatively, as we did in this work, we can identify and remove those features that contribute the most to the between-feature correlations.

All models implemented in this study were multicollinearity resistant except the linear model [60] which therefore required additional measures to avoid overfitting (Lines 293-301). In the linear model, in order to minimize the effect of collinearity, solve the issue of the number of predictor variables being greater than the sample size, estimate all parameters including the constant, and finally be able to fit the overall model during the training stage, we opted to reduce the number of predictors by using an algorithm to remove a subset of those features involved with the most high pairwise correlations such that the sample (training set) size is two more than the number of predictors (allowing for a residual degree of freedom), and all of the remaining pairwise Pearson correlation coefficients are below a 0.90 threshold [60].

[Note: HTML markup is below. Please do not edit.]

Reviewers' comments:

Reviewer's Responses to Questions

Comments to the Author

1. If the authors have adequately addressed your comments raised in a previous round of review and you feel that this manuscript is now acceptable for publication, you may indicate that here to bypass the “Comments to the Author” section, enter your conflict of interest statement in the “Confidential to Editor” section, and submit your "Accept" recommendation.

Reviewer #1: (No Response)

Reviewer #2: All comments have been addressed

2. Is the manuscript technically sound, and do the data support the conclusions?

The manuscript must describe a technically sound piece of scientific research with data that supports the conclusions. Experiments must have been conducted rigorously, with appropriate controls, replication, and sample sizes. The conclusions must be drawn appropriately based on the data presented.

Reviewer #1: No

Reviewer #2: Yes

---

3. Has the statistical analysis been performed appropriately and rigorously?

Reviewer #1: No

Reviewer #2: Yes

---

4. Have the authors made all data underlying the findings in their manuscript fully available?

The PLOS Data policy requires authors to make all data underlying the findings described in their manuscript fully available without restriction, with rare exception (please refer to the Data Availability Statement in the manuscript PDF file). The data should be provided as part of the manuscript or its supporting information, or deposited to a public repository. For example, in addition to summary statistics, the data points behind means, medians and variance measures should be available. If there are restrictions on publicly sharing data—e.g. participant privacy or use of data from a third party—those must be specified.

Reviewer #1: No

Reviewer #2: Yes

---

5. Is the manuscript presented in an intelligible fashion and written in standard English?

PLOS ONE does not copyedit accepted manuscripts, so the language in submitted articles must be clear, correct, and unambiguous. Any typographical or grammatical errors should be corrected at revision, so please note any specific errors here.

Reviewer #1: Yes

Reviewer #2: Yes

---

6. Review Comments to the Author

Please use the space provided to explain your answers to the questions above. You may also include additional comments for the author, including concerns about dual publication, research ethics, or publication ethics. (Please upload your review as an attachment if it exceeds 20,000 characters)

Reviewer #1: The authors didn't address my concerns about the model overfitting issue at all. Instead of rebuttal using statement in the manuscript (which have no proof/reference, such as '15-day intervals and averages reduced the correlation between inputs'), please provide statistical evidence that your model is not overfitting, such as training accuracy and testing accuracy when you do machine learning modeling. or at least provide "adjusted R-square" which is adjusted for the number of parameters, or ANOVA test to proof the independence of your inputs, or at least correlation scatter plots between inputs. But any way, 24 parameters

for 23 observations are way too many to avoid overfitting. Please read this post by Jim Frost for reference https://statisticsbyjim.com/regression/overfitting-regression-models/.

R: We thank the Reviewer as his remarks helped us better rephrase and explain how we prepared the data and implemented the models (Lines 293-301). All models implemented in this study were multicollinearity resistant except the linear model [60] and measures to avoid overfitting were applied. For instance, the "one standard error" rule of Breiman et al. [61] was used, and the methods' built-in features were invoked to automatically select features, tune hyperparameters to the data set, and select the best final model for the downstream validation step. In the process of data preparation, zero-variance features were removed and those remaining were centered and scaled in order to avoid features with zero or near-zero variance which can behave like second intercepts [63]. In the linear model, in order to minimize the effect of collinearity, solve the issue of the number of predictor variables being greater than the sample size, estimate all parameters including the constant, and finally be able to fit the overall model during the training stage, we opted to reduce the number of predictors by using an algorithm to remove a subset of those features involved with the most high pairwise correlations such that the sample (training set) size is two more than the number of predictors (allowing for a residual degree of freedom), and all of the remaining pairwise Pearson correlation coefficients are below a 0.90 threshold [60].

Several metrics of model performance were provided throughout the manuscript e.g., in Figures 5 and 7 for the model calibration stage, in Figures 6 and 8 and Table 3 for the validation stage.

Reviewer #2: Authors replied to my comments, improved their manuscript and I have no further comments.

---

7. PLOS authors have the option to publish the peer review history of their article (what does this mean?). If published, this will include your full peer review and any attached files.

If you choose "no", your identity will remain anonymous but your review may still be made public.

Do you want your identity to be public for this peer review? For information about this choice, including consent withdrawal, please see our Privacy Policy.

Reviewer #1: No

Reviewer #2: No

[NOTE: If reviewer comments were submitted as an attachment file, they will be attached to this email and accessible via the submission site. Please log into your account, locate the manuscript record, and check for the action link "View Attachments". If this link does not appear, there are no attachment files.]

While revising your submission, please upload your figure files to the Preflight Analysis and Conversion Engine (PACE) digital diagnostic tool, https://pacev2.apexcovantage.com/. PACE helps ensure that figures meet PLOS requirements. To use PACE, you must first register as a user. Registration is free. Then, login and navigate to the UPLOAD tab, where you will find detailed instructions on how to use the tool. If you encounter any issues or have any questions when using PACE, please email PLOS at figures@plos.org. Please note that Supporting Information files do not need this step.
