# Supplementary material for: Machine learning models based on remote and proximal sensing as potential methods for in-season biomass yields prediction in commercial sorghum fields
Source: PLoS One. 2021 Mar 25;16(3):e0249136. doi: 10.1371/journal.pone.0249136 (PMC7993797; doi:10.1371/journal.pone.0249136)
Supplement: Attachment — Submitted filename: Response to Reviewers.docx [file pone.0249136.s002.docx]

**Response to reviewers**

Dear Editor,

Thank you very much for considering my paper for publication in the journal PLOS ONE.

It’s a pleasure for myself and on behalf of the co-author, to be able use the Editor and Reviewer constructive comments and suggestions to improve this manuscript.

I systematically went through the comments and suggestions, providing our comments, answers, and actions we took in response to Editor and Reviewers concerns.

Below, our answers are provided in italicized font preceded with the capital letter “R” followed by a column “:”

Cordially.

Ephrem.

-------------------

PONE-D-20-28051
Machine Learning Models Based on Remote and Proximal Sensing as Potential Methods for In-Season Biomass Yields Prediction in Commercial Sorghum Fields
PLOS ONE

1. Please ensure that your manuscript meets PLOS ONE's style requirements, including those for file naming.

*R: Style and file naming were corrected according to PLOS ONE standard.*

 2. In your Methods section, please provide additional location information of the study sites, including geographic coordinates for the data set if available.

*R: Geographic coordinates were added to Table 1*

3. In your Methods section, please provide additional information regarding the permits you obtained for the work. Please ensure you have included the full name of the authority that approved the field site access and, if no permits were required, a brief statement explaining why.

*R: On lines 146-149 in the Manuscript file a sentence was added to explain there were formal contracts that regulated the trials in private commercial fields.*

 4. Thank you for stating the following after the Conclusion Section of your manuscript:

'Funding: This research was funded by the European Union, grant number 732064 (H2020-ICT-2016-1-innovation action and the APC was funded by the European Union through the project Data-driven Bioeconomy ([www.databio.eu](http://www.databio.eu)) and the Ministero delle Politiche Agricole, Alimentari, Forestali e del Turismo (Rome, Italy) through the project Risorse GeneticheVegetali (RGV/FAO) 2014–2019.'

We note that you have provided funding information that is not currently declared in your Funding Statement. However, funding information should not appear in the Acknowledgments section or other areas of your manuscript. We will only publish funding information present in the Funding Statement section of the online submission form.

a. Please remove any funding-related text from the manuscript and let us know how you would like to update your Funding Statement. Currently, your Funding Statement reads as follows:

'E.H. received funds from the European Union, grant number 732064 (H2020-ICT-2016-1-innovation action. The funders had no role in study design, data collection and analysis, decision to publish, or preparation of the manuscript.'

*R: As suggested, funding-related information was removed from the manuscript.*

 b. Please include your amended statements within your cover letter; we will change the online submission form on your behalf.

*R: Funding statements were included in the cover letter as suggested.*

 5. We note that Figure 1 in your submission contains map/satellite images which may be copyrighted.

All PLOS content is published under the Creative Commons Attribution License (CC BY 4.0), which means that the manuscript, images, and Supporting Information files will be freely available online, and any third party is permitted to access, download, copy, distribute, and use these materials in any way, even commercially, with proper attribution. For these reasons, we cannot publish previously copyrighted maps or satellite images created using proprietary data, such as Google software (Google Maps, Street View, and Earth). For more information, see our copyright guidelines: <http://journals.plos.org/plosone/s/licenses-and-copyright>.

 We require you to either (a) present written permission from the copyright holder to publish these figure specifically under the CC BY 4.0 license, or (b) remove the figure from your submission:

 a. You may seek permission from the original copyright holder of Figure 1 to publish the content specifically under the CC BY 4.0 license.

We recommend that you contact the original copyright holder with the Content Permission Form ([http://journals.plos.org/plosone/s/file?id=7c09/content-permission-form.pdf](about:blank)) and the following text:

“I request permission for the open-access journal PLOS ONE to publish XXX under the Creative Commons Attribution License (CCAL) CC BY 4.0 ([http://creativecommons.org/licenses/by/4.0/](about:blank)). Please be aware that this license allows unrestricted use and distribution, even commercially, by third parties. Please reply and provide explicit written permission to publish XXX under a CC BY license and complete the attached form.”

Please upload the completed Content Permission Form or other proof of granted permissions as an "Other" file with your submission.

*R: This figure was properly cited in the manuscript. It was originally published in Agronomy (Agronomy 2019, 9(4), 203;* [*https://doi.org/10.3390/agronomy9040203*](https://eur04.safelinks.protection.outlook.com/?url=https%3A%2F%2Fdoi.org%2F10.3390%2Fagronomy9040203&data=04%7C01%7Cephrem.habyarimana%40crea.gov.it%7C9c19ea130d2e47324b4c08d8a205faaf%7Cd59c04b9bde247f7b1b80be26a568618%7C0%7C0%7C637437494737474636%7CUnknown%7CTWFpbGZsb3d8eyJWIjoiMC4wLjAwMDAiLCJQIjoiV2luMzIiLCJBTiI6Ik1haWwiLCJXVCI6Mn0%3D%7C2000&sdata=UWfpC4F3KKSz38nyL7Y8sftNHB%2B5h6ZOFygufM3xls4%3D&reserved=0)*) under (CC BY) license (*[*http://creativecommons.org/licenses/by/4.0/*](https://eur04.safelinks.protection.outlook.com/?url=http%3A%2F%2Fcreativecommons.org%2Flicenses%2Fby%2F4.0%2F&data=04%7C01%7Cephrem.habyarimana%40crea.gov.it%7C9c19ea130d2e47324b4c08d8a205faaf%7Cd59c04b9bde247f7b1b80be26a568618%7C0%7C0%7C637437494737474636%7CUnknown%7CTWFpbGZsb3d8eyJWIjoiMC4wLjAwMDAiLCJQIjoiV2luMzIiLCJBTiI6Ik1haWwiLCJXVCI6Mn0%3D%7C2000&sdata=eOv5eXgviPpMmKO78W98h3fci0XZEOrqhxgqj7Wijqg%3D&reserved=0)*) which permits unrestricted use. Since the original paper was authored by myself and with a license that permits unrestricted use, there is no need for a written permission to copyright owner. These are the rules for the articles published in MDPI journals:
copyright is retained by the authors (*[*https://www.mdpi.com/authors/rights*](https://eur04.safelinks.protection.outlook.com/?url=https%3A%2F%2Fwww.mdpi.com%2Fauthors%2Frights&data=04%7C01%7Cephrem.habyarimana%40crea.gov.it%7C9b3ad847acfc49daebd908d8a267f5ac%7Cd59c04b9bde247f7b1b80be26a568618%7C0%7C0%7C637437914678200770%7CUnknown%7CTWFpbGZsb3d8eyJWIjoiMC4wLjAwMDAiLCJQIjoiV2luMzIiLCJBTiI6Ik1haWwiLCJXVCI6Mn0%3D%7C1000&sdata=xmSoVWUxTwULDYwHSB6cmV801TpCf%2Fl0P8NKpjseIwQ%3D&reserved=0)*).*

In the figure caption of the copyrighted figure, please include the following text: “Reprinted from [ref] under a CC BY license, with permission from [name of publisher], original copyright [original copyright year].”

*R: The text was added in the figure caption of the copyrighted Fig 1.*

 6. Please include captions for your Supporting Information files at the end of your manuscript, and update any in-text citations to match accordingly. Please see our Supporting Information guidelines for more information: [http://journals.plos.org/plosone/s/supporting-information](about:blank)

*R: The captions of the S1 Table was added at the end of the manuscript as suggested.*

Additional Editor Comments:

Thank you for your submission and my apologies for delays caused by COVID-19. I have considered the input of both reviewers, whom I respect and value greatly. R2 brings up the point of small sample size in testing, and you do address that in your discussion. R1 brings up points about your modelling efforts and how to better clarify those efforts. Addressing those and other concerns they both raise will be helpful. To R1's point on small sample size--this is concerning, but I think framing this paper more as a proof of concept (which you do at points) would be more advisable. Your introduction reads like a review. I think carefully paying attention to where you can augment the existing text to make sure readers understand this is an attempt to push current methods towards opening up new avenues of research would be helpful. Thank you.

*R: Small sample size: this issue was addressed in the manuscript particularly on lines 467-479 of the manuscript.*


[Note: HTML markup is below. Please do not edit.]

Reviewers' comments:

Reviewer's Responses to Questions

**Comments to the Author**

1. Is the manuscript technically sound, and do the data support the conclusions?

The manuscript must describe a technically sound piece of scientific research with data that supports the conclusions. Experiments must have been conducted rigorously, with appropriate controls, replication, and sample sizes. The conclusions must be drawn appropriately based on the data presented.

Reviewer #1: Yes

Reviewer #2: No

2. Has the statistical analysis been performed appropriately and rigorously?

Reviewer #1: Yes

Reviewer #2: No

3. Have the authors made all data underlying the findings in their manuscript fully available?

The [PLOS Data policy](https://eur04.safelinks.protection.outlook.com/?url=http%3A%2F%2Fwww.plosone.org%2Fstatic%2Fpolicies.action%23sharing&data=04%7C01%7Cephrem.habyarimana%40crea.gov.it%7Cacc3019907c74ffce46808d8a11c564e%7Cd59c04b9bde247f7b1b80be26a568618%7C0%7C0%7C637436491162892551%7CUnknown%7CTWFpbGZsb3d8eyJWIjoiMC4wLjAwMDAiLCJQIjoiV2luMzIiLCJBTiI6Ik1haWwiLCJXVCI6Mn0%3D%7C1000&sdata=UAmHajZByy%2B6p2Z4esdcLIH8ZwsFDghjpYHkrhDR6lg%3D&reserved=0) requires authors to make all data underlying the findings described in their manuscript fully available without restriction, with rare exception (please refer to the Data Availability Statement in the manuscript PDF file). The data should be provided as part of the manuscript or its supporting information, or deposited to a public repository. For example, in addition to summary statistics, the data points behind means, medians and variance measures should be available. If there are restrictions on publicly sharing data—e.g. participant privacy or use of data from a third party—those must be specified.

Reviewer #1: Yes

Reviewer #2: Yes

4. Is the manuscript presented in an intelligible fashion and written in standard English?

PLOS ONE does not copyedit accepted manuscripts, so the language in submitted articles must be clear, correct, and unambiguous. Any typographical or grammatical errors should be corrected at revision, so please note any specific errors here.

Reviewer #1: Yes

Reviewer #2: Yes

5. Review Comments to the Author

Please use the space provided to explain your answers to the questions above. You may also include additional comments for the author, including concerns about dual publication, research ethics, or publication ethics. (Please upload your review as an attachment if it exceeds 20,000 characters)

Reviewer #1: The manuscript compared several crop yield models with several different remotely sensed optical features in several sorghum fields in Italy. It's an interesting topic and fit to the scope of PLOS ONE. While I have several concerns before the consideration of the publication:

My main concerns are about the feature selections used in learning model:
1.1. there is no description or discussion on how these features were selected

*R: The process of selecting the features used in the models was explained in the Manuscript particularly on lines 246-257, 259-267, and 289-300.*

1.2. there are 24 features/inputs in the model, while most of them are same features, like 14 features are fAPAR that captured in different time, please add more information about how did you handle the correlations between these inputs to avoid model overfitting

*R:* *Collecting data at 15-day intervals and averages reduced the correlation between inputs. On the other hand, several other steps were taken to avoid model overfitting and this was explained in the manuscript e.g., (1) Our work is a proof of concept accommodating small sample size, and, among models we included low-complexity models such as Bayesian ridge regression (Manuscript lines 274-275, 468-473), (2) implementing the “one standard error rule” and using fortnightly data (Manuscript lines 467-473).*

1.3. The description of different learning models is inadequate, more details are needed to show the differences of these learning models and why they are used in this study

*R: The models were comprehensively explained in the manuscript, particularly in lines 263-278, 471-480.*

1.4. Section 3.1 should be in methodology as it's the description of model inputs

*R: Section 3.1 “Descriptive statistics of the features used in modelling” present the analytics outcome and should therefore remain under Results’ section.*

Reviewer #2: This manuscript reports the result of applying different machine learning techniques for Sorghum biomass Prediction. Authors analysed different Sentinel-2 images and collected ground data from different fields using hand held sensors for chlorophyll and NDVI measurements from 2 different years. In general, the topic is current, interesting and the manuscript has adequate information for methodology and results. However, the ground observation number is not suitable for such analysis considering the different varieties, years and fields. Moreover, I have the following specific comments:

*R: The ground observation number can be considered suitable as this work is a proof of concept (Manuscript lines 468-469) aiming mainly at opening new research venues (lines 123-125). In addition, small-sized samples are expected to be a rule not an exception in agricultural sciences, particularly at demonstration or pilot levels requiring commercial fields (lines 592-593).*


L40: This sentence does not add value. It is just defining the word forecast by using the same word. Please revise.

*R: the sentence was edited (Manuscript lines 41-42)*

L41: (simplifies), I suggest to replace it with the word support.

*R: replaced as suggested (Manuscript line 42)*

L71: I don't think that it is possible to estimate corn yield 4 months before harvesting. Please revise this information.

*R: The Reviewer was right. There were typos in previous version, we fixed the errors as shown on lines 71-74 of the manuscript.*

L228: How did you apply the PROSAIL model to retrieve the fAPAR?

*R: We edited the text for a better reading, and the use of PROSAIL model was better described in the manuscript on lines 228-245.*

L252-261: Move this part to your introduction section.

*R: since this are short specific descriptions of the algorithms used in the work, we consider they should remain under Materials and methods”.*

L280: The number of observations is very low. I suggest to collect more ground observations as it is not possible to depend on these current results.

*R: The ground observation number can be considered suitable as this work is a proof of concept (Manuscript lines 468-469) aiming mainly at opening new research venues (lines 123-125). In addition, small-sized samples are expected to be a rule not an exception in agricultural sciences, particularly at demonstration or pilot levels requiring commercial fields (lines 592-593).*

L285-306: This information is not related to your methodology and elaborating more in a well known information. I suggest reducing this part and moving it to the introduction section.

*R: since the information is about the description of the model evaluation metrics implemented in the study as can be seen in the produced tables and figures, the information should remain under Materials and methods.*

L336 Please define where to find this information.

*R: prior to arriving at the Table 2, the information in question would have been read in paragraph above.*

For all figures: Please maintain a consistent style for figure legends, X and Y labels and values.

*R: each figure is a different graphic representation and is therefore a different figure. Yet, we tried to be as consistent as we could.*

6. PLOS authors have the option to publish the peer review history of their article ([what does this mean?](https://eur04.safelinks.protection.outlook.com/?url=https%3A%2F%2Fjournals.plos.org%2Fplosone%2Fs%2Feditorial-and-peer-review-process%23loc-peer-review-history&data=04%7C01%7Cephrem.habyarimana%40crea.gov.it%7Cacc3019907c74ffce46808d8a11c564e%7Cd59c04b9bde247f7b1b80be26a568618%7C0%7C0%7C637436491162902545%7CUnknown%7CTWFpbGZsb3d8eyJWIjoiMC4wLjAwMDAiLCJQIjoiV2luMzIiLCJBTiI6Ik1haWwiLCJXVCI6Mn0%3D%7C1000&sdata=WaduZuzUodgi83c9u1PoDVbM2XGJhgqIml%2BWelWUpUA%3D&reserved=0)). If published, this will include your full peer review and any attached files.


If you choose “no”, your identity will remain anonymous but your review may still be made public.


**Do you want your identity to be public for this peer review?** For information about this choice, including consent withdrawal, please see our [Privacy Policy](https://eur04.safelinks.protection.outlook.com/?url=https%3A%2F%2Fwww.plos.org%2Fprivacy-policy&data=04%7C01%7Cephrem.habyarimana%40crea.gov.it%7Cacc3019907c74ffce46808d8a11c564e%7Cd59c04b9bde247f7b1b80be26a568618%7C0%7C0%7C637436491162902545%7CUnknown%7CTWFpbGZsb3d8eyJWIjoiMC4wLjAwMDAiLCJQIjoiV2luMzIiLCJBTiI6Ik1haWwiLCJXVCI6Mn0%3D%7C1000&sdata=tEe7qbFjUARv5gkMx0DKTQG0zaK4giD0svP0fYACmf0%3D&reserved=0).

Reviewer #1: No

Reviewer #2: No

[NOTE: If reviewer comments were submitted as an attachment file, they will be attached to this email and accessible via the submission site. Please log into your account, locate the manuscript record, and check for the action link "View Attachments". If this link does not appear, there are no attachment files.]

While revising your submission, please upload your figure files to the Preflight Analysis and Conversion Engine (PACE) digital diagnostic tool, [https://pacev2.apexcovantage.com/](https://eur04.safelinks.protection.outlook.com/?url=https%3A%2F%2Fpacev2.apexcovantage.com%2F&data=04%7C01%7Cephrem.habyarimana%40crea.gov.it%7Cacc3019907c74ffce46808d8a11c564e%7Cd59c04b9bde247f7b1b80be26a568618%7C0%7C0%7C637436491162912540%7CUnknown%7CTWFpbGZsb3d8eyJWIjoiMC4wLjAwMDAiLCJQIjoiV2luMzIiLCJBTiI6Ik1haWwiLCJXVCI6Mn0%3D%7C1000&sdata=TzNvLw2kuhCAYJl0Oaei9sRvCbmR%2F7ntk4s5qXzWlMA%3D&reserved=0). PACE helps ensure that figures meet PLOS requirements. To use PACE, you must first register as a user. Registration is free. Then, login and navigate to the UPLOAD tab, where you will find detailed instructions on how to use the tool. If you encounter any issues or have any questions when using PACE, please email PLOS at [figures@plos.org](mailto:figures@plos.org). Please note that Supporting Information files do not need this step.
